# Effects of Electroacupuncture at Head Points on the Function of Cerebral Motor Areas in Stroke Patients: A PET Study

**DOI:** 10.1155/2012/902413

**Published:** 2012-08-22

**Authors:** Zuo Fang, Jia Ning, Chang Xiong, Yao Shulin

**Affiliations:** ^1^TCM and Acupuncture & Moxibustion Department of Nanlou, Chinese PLA General Hospital, Beijing 100853, China; ^2^Department of Nosocomial Infection and Disease Control, Chinese PLA General Hospital, Beijing 100853, China; ^3^Chinamed USA, Charlottesville, VA 22901, USA; ^4^Department of Nucleus Medicine, Chinese PLA General Hospital, Beijing 100853, China

## Abstract

Positron emission tomography (PET) is used to observe the cerebral function widely and is a good method to explore the mechanism of acupuncture treatment on the central nervous system. By using this method, we observed the cerebral function of 6 patients suffering from ischemic stroke after receiving EA treatment at Baihui(GV20) and right Qubin(GB7). The results were: (1) the glucose metabolism changed significantly on primary motor area (M1), premotor cortex (PMC), and superior parietal louble (LPs) bilaterally, as well as the Supplementary Motor Area (SMA) on the unaffected hemisphere right after the first EA treatment. (2) The glucose metabolism on bilateral M1 and LPs changed significantly after three weeks of daily EA treatments. (3) The glucose metabolism on other areas such as insula, putamen, and cerebellum changed significantly. It demonstrated that EA at Qubin and Baihui couldactivate the cerebral structures related to motor function on the bilateral hemispheres.We concluded that EA was very helpful for the cerebral motor plasticity after the ischemic stroke. Also based on this study we assumed that the brain plasticity should be a network and that acupuncture participated in some sections of this course.

## 1. Introduction

Acupuncture has been used in stroke rehabilitation in China for over 3000 years. However, its mechanisms are still under discussion. Most researchers used biochemical or anatomical tests to observe the animals or electrophysiological tests to observe human beings and tried to explain the mechanisms [1–3]. Unfortunately what they got was just indirect evidence. PET (positron emission tomography), a vivo functional examination technology can be done without invasive procedures. Its image reflects not only the structure of the organs and tissues, but also their physiological and biochemical changes. It can clearly reflect the change of corresponding functional areas of brain after stimulus. Therefore PET is called “vivo molecular biological imaging” [4]. Since the functional changes can be detected visually, it has been widely used in the study of the brain function [5–7] and the central nervous mechanism of acupuncture effect [8, 9]. However, no research was done with involving movements. We believed that the study on the changes of glucose metabolism after EA with movements which could activate the cerebral motor functional areas could reflect impact of EA on motor function more accurately, compared with the study in the resting state after accepting the EA. 

EA has a nearly 200-year history. French physician Louis Berlioz was the first to apply the electricity on acupuncture needles in his clinical work in 1810. He believed that the current generated by the battery might enhance the therapeutic effect of the acupuncture needles [10]. In China, EA appeared for the first time in the Journal of Acupuncture and Moxibustion 1934 and Tang Shicheng proposed that EA with pulsed current generated by the electronic tube could be used clinically. It was probably Zhu longyu in Shaanxi Province, China who brought about the therapeutic EA officially in 1950s. He invented the EA stimulator successfully in 1953 and found that EA has a significant analgesic effect in 1956 [11]. Since then EA has been widely used in acupuncture anesthesia and other diseases treatment. After nearly 60 years of clinical and experimental applications, EA has become one of the most widely used medical therapeutic methods. It promoted the development of clinical and scientific research on acupuncture somehow. The amount of stimulation of EA is more objective than the traditional manual acupuncture, so it has been used in acupuncture clinical or experimental studies for control studies. A large number of studies have shown that EA has a significant treatment effect on stroke [12, 13], and in the department where I worked, EA was a routine clinic treatment for stroke. So we used EA in this study.

So far the researches on stroke recovery mechanisms have found (1) the recovery of an animal experimental focal cerebral ischemia (sensory or motor cortex) was related to the reorganization in the adjacent unaffected cortex around the lesion [14, 15]; (2) the activation of the supplementary motor area (SMA) is considered with the recovery of motor function [16]; (3) during the recovery process, a certain number of cells and tissues of the unaffected hemisphere change [17].

Therefore, we observed cerebral glucose metabolism in stroke patients before and after EA treatments with patients' fist-clenching movement, in order to explore the effect of head acupoints on multiple cerebral areas above mentioned and to interpret the mechanism of acupuncture on stroke patients' motor function recovery.

## 2. Patients and Methods

### 2.1. Patients

Six right-handed stroke patients (3 males, 3 females) were recruited for the study. Each patient received an explanation of the study protocol and signed an informed consent prior to participating in the study. Patients accepted short-term movement task training before the experiment.

Inclusion criteria were (i) first-ever ischemic stroke (confirmed by computerized tomography (CT) scan or magnetic resonance imaging (MRI)) involving the right basal ganglion region; (ii) age 50–75 years; (iii) admission within 1–3 months of onset; (iv) upper extremity motor function of patients was damaged, but patients could finish the movements that the study required (muscle strength was 3-4); (v) for the diabetic patients with the controlled blood sugar, the blood sugar was tested on the dates of the scan for the correction.

Patients were excluded if they: (i) had severe diabetes and severe heart disease and (ii) were taking any central nervous system depressants or stimulant drugs in the previous month.

### 2.2. Instrument and Methods

#### 2.2.1. Instrument: Positron Emission Tomography (PET)

PET scanner (ECAT EXACT HR^+^, Siemens Co. Germany) was used with the scanning mode of three dimensions (3D), and the thickness of each slice was 3 mm with septa retracted and scatters correction. The EXACT HR^+^ had a 15.2-cm axial FOV, sufficient to image the whole brain in a single scan.

#### 2.2.2. Methods

The temperature of the examination room was 22–24°C. The tracer was 18 fluoride-deoxyglucose (18F-FDG, produced by accelerator CTIRDS111, and its purity >95%, 148–185 MBq), which was administered by intravenous injection at 110 mCi/kg (bodyweight). The patients had the earplugs and eye patches on to block auditory and visual stimuli until the end of the scan. Before each scan the patient's head was placed into a supporting device, localized by laser, and fixed in a position where superior and inferior laser lines were parallel to the orbit mastoid (OM) line and the cerebrum and the cerebellum were covered.

#### 2.2.3. Procedure Details

**Figure 1:**
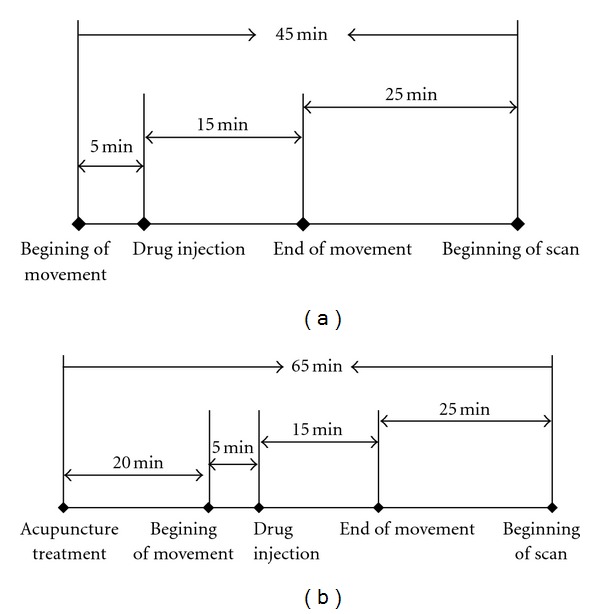
(a) First test chart flow diagram. (b) Second test chart flow diagram.

**Figure 2:**
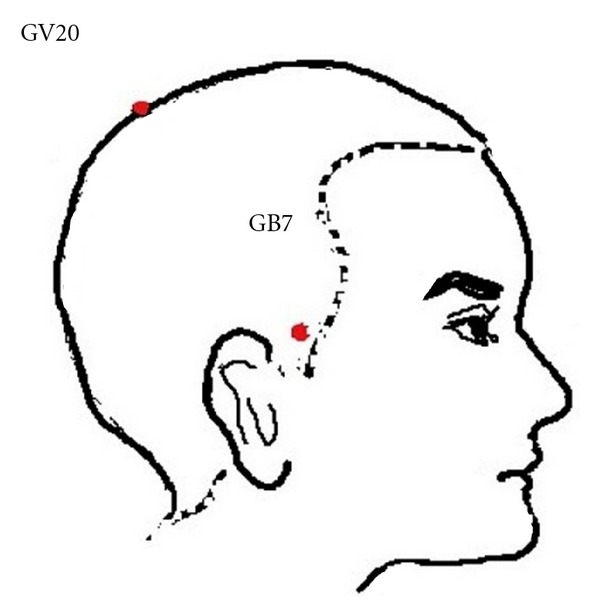
Schematic diagram of GV20 and GB7.

The observation was conducted as the followed steps. (1) The patient laid flat on the examination table and made fist-clenching movements regularly (the affected hand) at a frequency of about 0.5 Hz (metronome interrupter, Nikko, P1440, Japan) continuously for 20 minutes. Five minutes after the movements started, 18 fluorine deoxyglucose (18F-FDG) (4mci) was injected into the vein of the unaffected hand. 40 minutes after the injection, the patients had the PET scans (Figure 1(a)). (2) On the following day, the patient accepted acupuncture treatment at Baihui (GV20) and right Qubin (GB7, on the affected hemisphere) (Figure 2) for 20 minutes. The needles were connected to an electroacupuncture (EA) therapeutic apparatus. Then the same procedure as the first step was repeated, the images of the second time were collected (Figure 1(b)). (3) After three weeks of EA daily treatment the patients got the same PET scan as the first step. 

Needling process: the acupuncture pointes were cleaned with 75 percent alcohol. The needles were the single-use, disposable stainless steel acupuncture needles (Huatuo, Suzhou Medical Supplies Co. Ltd; Suzhou, China), with a diameter of 0.25 mm and a length of 25 mm. The needles were inserted horizontally into both acupuncture points, forming a less than 30 angle with the skin surface. Depth of needle insertion was approximately 10 mm. Then two needles were connected to a commercial electro-acupuncture device (model SDZ-V, Suzhou Medical Supplies Co. Ltd; Suzhou, China). The frequency was 2 Hz with continuous waves. The intensity of the stimulation was increased to the point where the patient reported the needling reaction and then it was adjusted gradually to a comfortable intensity and remained at that level for 20 minutes.

#### 2.2.4. Image Analysis

PET data were processed and analyzed by statistical parametric mapping (SPM99) (Institute of Neurology, University of London, London, UK) and the Matlab 6.1 program (Mathworks Inc., Sherborn, MA, USA) was used. Brain images were anatomically normalized to a standard brain template (FDG-PET version adapted to the MNI-MRI template by Montreal Neurological Institute) by linear (affine) and nonlinear transformations to minimize intersubject anatomical variations by using an SPM routine. To identify brain regions in which the perfusion and glucose metabolism had changed following EA, linear contrasts were used to test for regionally specific differences between groups, producing paired *t*-statistic maps in Talairach standard space. These *t*-statistics were transformed to corresponding Z maps, which constituted the statistical map (SPM-Z). The peak voxel-based significance of statistics was set at uncorrected *P* < 0.05.

## 3. Results

**Figure 3:**
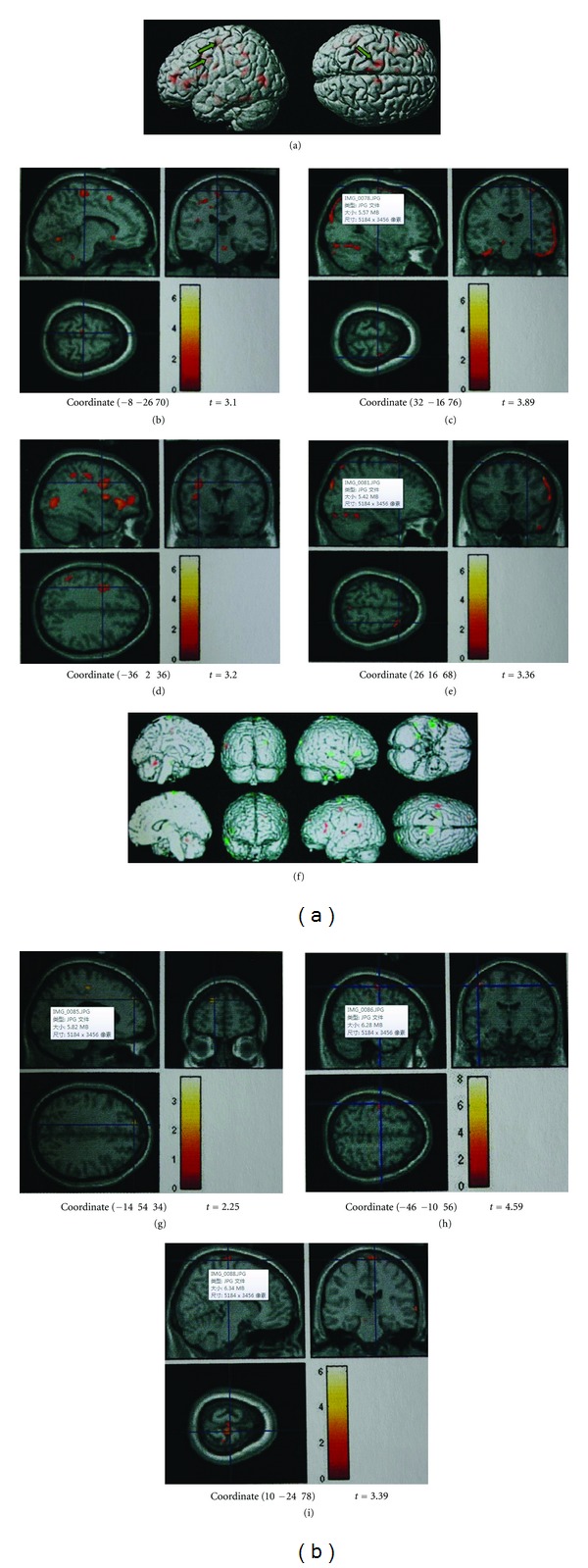
(a) EA instant effect: brain glucose metabolism of the stroke patients increases. The red represented the areas with increased glucose metabolism, and the green arrow referred to M1. (b) The glucose metabolism on the left precentral gyrus increased. (c) The glucose metabolism on the right precentral gyrus decreased. (d) The glucose metabolism on the left midfrontal gyrus increased. (e) The glucose metabolism on the right midfrontal gyrus decreased. (f) Three weeks of EA treatment effect: brain glucose metabolism alteration. The red represented the areas with increased glucose metabolism, and the green represented the areas with decreased glucose metabolism. The glucose metabolism alteration areas included bilateral M1, temporal lobe, and so forth. (g) Left superior frontal gyrus glucose metabolism increased. (h) Left precentral gyrus glucose metabolism increased. (i) Right precentral gyrus glucose metabolism decreased.

 (1) EA instant effects: the significant increase of glucose metabolism was found on the unaffected side: the Primary motor area (M1), the precentral gyrus (the 4th Area), the supplementary motor area (SMA), the medial frontal gyrus (the 6th area), premotor cortex (PMC), the central frontal gyrus (the 6th Area), and the superior parietal lobule (LPs, the 7th Area) (Table 1(a), Figures 3(a), 3(b), and 3(d)). The decrease of glucose metabolism was found on M1, PMC, and LPs in the affected side (Table 1(b), Figures 3(c) and 3(e)).

(2) After three weeks daily EA treatments, the increase of glucose metabolism (Table 2(a), Figure 3(f), Figures 3(g), and 3(h)) was found on the unaffected hemisphere (left side); and the decrease of glucose metabolism (Table 2(b), Figures 3(f) and 3(i)) was found in the affected hemisphere. Among these areas, M1 and LPs were related to motor function.

(3) Besides these areas related to motion directly, the other areas where glucose metabolism had changed included middle temporal gyrus, superior temporal gyrus, putamen, and cerebellum.

## 4. Discussion

(1) *Huang Di nei jing su wen* pointed out that the *Head is the Smart House*. It means that the human's meridian Qi concentrates in the head and face through meridians and branched channels. It also pointed out that *Qi goes out of the brain*. It means that the driving force comes from the brain. Therefore, the limbs are controlled by the brain. Motor dysfunction, hemiplegia, is the main symptom of stroke. And the location of this disease is in the brain. 

In this study, head acupoints took the role of local treatments. Furthermore, it is very important to select the points on Governor and Foot Gallbladder meridians to treat stroke. Since the Governor meridian goes through the spine upto the neck and brain and is the governor of the Yang of the body; and the Foot Gallbladder meridian goes through the body side up to the top of the head and its meridian sinew intercrosses the musculature of meridians on two sides of the body, and goes along with the Yang Heel Vessel. Their main function is to treat paralysis after stroke and all other kinds of symptoms in the head. Previous research showed that the acupuncture treatments at those points alleviated brain damage after ischemic stroke in the monkeys and rats [18, 19]. And some studies demonstrated that the GV20 is located in the area of the frontal lobe of the anterior precentral sulcus [20]. So we selected these two points. Based on traditional Chinese medical theories, acupuncture on these two points can activate Qi of the Governor and Gallbladder meridians, regulate the channels' function, and balance Yin and Yang of the body, thus contribute to the improvement of motor function of limbs. Other previous clinical studies have shown that: stimulating Baihui could well regulate central bioelectrical activity in cerebral cortex of stroke patients and was conducive to the resurrection of the nerve cells of the penumbra or awakening dormant brain cells. So the link between the functional areas of cerebral cortex and compensatory function were strengthened [21, 22].

**Figure 4:**
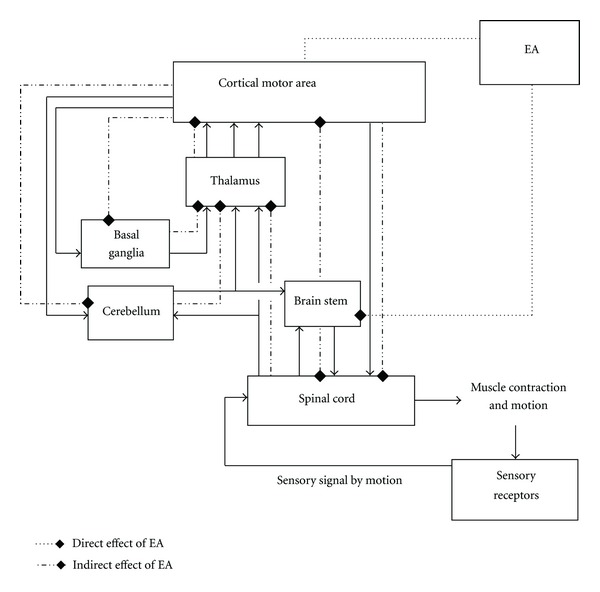
Schematic diagram of the relationship between the structures of the motor system.

(2) It helped us realize that noninvasive functional examination in vivo could clearly reflect the changes of biological metabolism of cerebral nervous tissue after acupuncture by using PET. Asking the patients to move their hands to activate or deactivate the glucose metabolism was helpful in identifying which cerebral motor areas were engaged and we could obtain the direct evidence from spatial and time aspects, which could reflect the effect of acupuncture on the motor recovery of stroke patients more objectively than in the resting condition.

(a) Instant effect of EA: the results of the study showed that after EA, the bilateral M1, PMC, and LPs, as well as SMA of the unaffected hemisphere had significant changes in glucose metabolism, with remarkable increase in these regions of the unaffected hemisphere and decrease in the affected hemisphere. Acupuncture activated not only the cerebral tissues of the affected hemisphere, but also the related regions of the unaffected hemisphere, particularly SMA. The results confirmed the previous research: the activation of the SMA was considered to be related to the recovery of the motor function [23, 24]. In humans with unilateral stroke, previous studies have found increased excitability [25] and, sometimes, increased cerebral blood flow in the contralateral cortex [26, 27]. Some of these contralesional changes are related to enhanced ipsilesional function. It demonstrated that acupuncture could activate bilateral motor areas of the brain and initiate excitement of the nerve tissue related to the motor activity and it played a role of compensation, which is important for the recovery of neural tissue of the semidark band and the activation of potential functional regions.

(b) Effect of three weeks of EA treatments: after three weeks of daily acupuncture treatments, the changes in the region related to the motor ability directly was only the M1, but still showed the same trend as the instant effects: bilateral changes with increased glucose metabolism on the unaffected hemisphere and decreased metabolism on the ipsilesional hemisphere. It showed that the contralateral hemisphere played an important role in the stroke recovery process [28, 29] and that EA treatment for stroke patients had played a good role in the recovery of motor function.

(3) We adopted a functional description to describe the experimental results since different cerebral structures often have the same function. M1 is in the precentral gyrus and paracentral lobule, equivalent to Brodmann area 4 and controls voluntary movements. PMC is located in Area 6 in the front of the precentral gyrus. SMA mainly is located in area 6 in the internal and upper dorsolateral hemisphere. They are related to the state of readiness before exercises. This function division indicated that the region of programming the movement preparation and controlling the movement implementation cannot be limited to a special area and may involve a lot of “modules” in a dynamic network. After EA treatment, these areas had the metabolic changes. It demonstrated that the EA on head acupoints may improve the recovery of motor function through the regulation of “module” of this network.

 (4) After EA at Qubin (GB7) and Baihui (GV20), the changes took place not only in the motor areas but also in the insular cortex, temporal lobe, occipital lobe, putamen, and cerebellum. These changes may be related to the specificity of the acupoints. However, some literatures also mentioned that metabolic changes on the areas such as the insular cortex, parietal lobe, thalamus, putamen, and cerebellum were relevant to the movement [30, 31]. In the central nervous system, no functional areas are simple and independent. Only motor function is involving multiple areas (Figure 4 [32]). These areas connected closely with ipsilateral and contralateral cortex or nuclei and interacted with each other by both excitatory and inhibitory mechanisms. It is a complex network. So the metabolic function changes in these areas were most likely indirect evidence of the changes of motor function. We believed that EA at head acupoints may activate all levels of the structures related to the motor function (Figure 4): EA at Baihui (GV20) and Qubin (GB7) may affect the structure of motion system in two ways directly or indirectly at all levels. One way was that the stimuli went up through the brain stem, cornuposterius medullae—trigeminal lemniscus, spinothalamic tract—thalamus—cerebral cortex, and interacted between the cerebral cortices and influenced on subcortical structures. The other possible way was that EA at head acupoints may produce weaker bioelectrical signals in local neuromuscular tissue, thereby creating a weaker biological magnetic field and penetrating the skull, directly to influence the cerebral cortex and its associated areas [33] and then to adjust movement structure at all levels through the neural network. This presumption needed further experimental study.

(5) The increased or decreased changes of glucose metabolism after acupuncture reflected the degrees of the excitement and inhibition of the related cerebral areas. Those excitement and inhibition had the important role. Because the neurons have an extensive mutual connection, they may excite or inhibit each other and serve to conduct sequential processing and transmission of the complicated information. The inhibition of some neurons may be the aftereffect of excitement of some other neurons or the initiation of the excitement in other sites. The metabolic decrease in some cerebral areas was likely a compensative mode of other areas. Hence, we should pay more attention to which cerebral areas were involved and how much changes happened. When acupuncture activated some areas, it also induced changes in relative areas. It can be thought that the regulation of acupuncture should be a relatively specific network effect and a multiple regulation course.

In conclusion, EA at head points might activate the cerebral motor areas bilaterally and induce the excitation of nerve tissue related to motion, and the activation of other regions demonstrated that the reorganization of the injured motor function was a neural network behavior, and that acupuncture may act on multiple aspects of the neural network, thus further contributing to the recovery of motor function. 

## Figures and Tables

**Table 1a:** (a)

	Peak	Corrected	Coordinates		
	*t* value	*P* value	*x*	*y*	*z*
GPrC (Area4) L	3.10	0.0087	−8	−26	70
GFd (Area6) L	2.99	0.0101	−16	−12	58
LPs (Area7)L	2.79	0.0134	−32	−42	50
GFm (Area6) L	3.20	0.0075	−36	2	36
GFd (Area6) R	3.05	0.0092	10	−14	56
GTm (Area37) L	5.91	<10^−4^	−44	−68	8
GTs (Area22) L	3.75	0.0036	−54	−48	18
Cerebellum L	3.51	0.0049	−8	−66	−10
Putamen L	3.01	0.0097	−12	16	−4

*P* < 0.05, and the coordinates indicated the location of maximally significant activity.

**Table 1b:** (b)

	Peak	Corrected	Coordinates		
	*t* value	*P* value	*x*	*y*	*z*
GPrC (Area4) R	3.89	0.0030	32	−16	76
GFm (Area6) R	3.36	0.0060	26	16	68
LPs (Area7) R	2.23	0.0302	2	−60	68
GFd (Area9) R	4.17	0.0021	6	62	26
GFm (Area10) R	2. 51	0.0200	48	54	4
GTm (Area21) R	5.38	<10^−4^	60	0	−26
GFm (Area6) L	2.39	0.0236	−34	10	60

*P* < 0.05, and the coordinates indicated the location of maximally significant activity.

**Table 2a:** (a)

	Peak	Corrected	Coordinates		
	*t* value	*P* value	*x*	*y*	*z*
GPrC (Area4) L	4.59	0.0029	−46	−10	56
GFm (Area6) L	2.68	0.0217	−68	18	20
LPs (Area7) L	2.25	0.0291	−14	54	34
GFd (Area9) L	3.75	0.0036	−54	−50	22
Cerebellum R	3.14	0.0082	28	−40	−50

*P* < 0.05, and the coordinates indicated the location of maximally significant activity.

**Table 2b:** (b)

	Peak	Corrected	Coordinates		
	*t* value	*P* value	*x*	*y*	*z*
GPrC (Area4) R	3.39	0.0058	10	−24	78
GFd (Area45) R	2.72	0.0147	50	28	4
thalamus R	3.11	0.0085	20	−10	12
GTs (Area22) R	2.95	0.0107	74	−26	4
GTm (Area21) R	2.80	0.0131	60	−2	−20
Cuneus (Area19) R	2.61	0.0173	24	−90	30
GTs (Area38) R	1.96	0.0441	50	16	−10
GFs (Area6) L	2.39	0.0238	−26	−8	70

*P* < 0.05, and the coordinates indicated the location of maximally significant activity.
